# An attempt to dissect a peripheral marker based on cell pathology in Parkinson's disease

**DOI:** 10.1007/s00702-021-02364-6

**Published:** 2021-06-09

**Authors:** Francesca Biagioni, Rosangela Ferese, Filippo Sean Giorgi, Nicola Modugno, Enrica Olivola, Paola Lenzi, Stefano Gambardella, Diego Centonze, Stefano Ruggieri, Francesco Fornai

**Affiliations:** 1 I.R.C.C.S Neuromed, Via Atinense 18, 86077 Pozzilli, IS Italy; 2 Department of Translational Research and New Technologies in Medicine and Surgery, University of Pisa, Via Roma 55, 56126 Pisa, Italy; 3 Department of Biomolecular Sciences, University of Urbino “Carlo Bo”, Urbino, Italy; 4 Department of Systems Medicine, Tor Vergata University, Rome, Italy

**Keywords:** Parkinson’s disease, Peripheral blood mononuclear cells, Autophagy, Synuclein, Vacuoles, LC3

## Abstract

Peripheral markers in Parkinson’s disease (PD) represent a hot issue to provide early diagnosis and assess disease progression. The gold standard marker of PD should feature the same reliability as the pathogenic alteration, which produces the disease itself. PD is foremost a movement disorder produced by a loss of nigrostriatal dopamine innervation, in which striatal dopamine terminals are always markedly reduced in PD patients to an extent, which never overlaps with controls. Similarly, a reliable marker of PD should possess such a non-overlapping feature when compared with controls. In the present study, we provide a novel pathological hallmark, the autophagosome, which in each PD patient was always suppressed compared with each control subject. Autophagosomes were counted as microtubule-associated proteins 1A/1B light chain 3B (LC3)-positive vacuoles at ultrastructural morphometry within peripheral (blood) blood mononuclear cells (PBMC). This also provides the gold standard to assess the autophagy status. Since autophagy may play a role in the pathogenesis of PD, autophagosomes may be a disease marker, while participating in the biology of the disease. Stoichiometric measurement of α-synuclein despite significantly increased in PD patients, overlapped between PD and control patients. Although the study need to be validated in large populations, the number of autophagy vacuoles is neither related with therapy (the amount was similarly suppressed in a few de novo patients), nor the age in PD or controls.

## Introduction

The search for early peripheral markers in Parkinson’s disease (PD) is a hot topic in neurological research during the past three decades. The occurrence of altered dopamine (DA) levels and metabolites in the blood and cerebrospinal fluid (CSF) has been intensely investigated, although it is considered inconclusive due to the remote site (blood and CSF) where the samples were collected compared with the affected brain area placed in the brainstem. This makes unreliable the amount of catecholamine as a marker of the integrity of the nigrostriatal system, due to the variety of biochemical steps taking place on monoamine and metabolites in the move from the CNS to distant peripheral sites (Kopin 1985; Eisenhofer et al. 2004). Imaging techniques providing a molecular detection of the nigrostriatal DA innervation targeting the DA transporter or DOPA-decarboxylase cannot be carried out routinely to predict PD during pre-clinical stages, while providing an information, which has been defined as a succedaneum concerning the integrity of the nigrostriatal system (de la Fuente-Fernández 2012). The progress in the neurobiology of disease moved recently to focus the search of disease markers considering the proteinopathy which takes place in PD. Thus, a number of studies focused on the main protein alteration which occurs in PD patients concerning α-synuclein (α-syn). Several studies in the last two decades probed the amount of α-syn in the blood, CSF and other fluids including saliva as a feasible marker in PD (Vivacqua et al. 2016; Wang et al. 2015). These studies became more and more elaborated in detecting specific protein conformation and providing a significant correlation between PD and specific isoform of α-syn in various fluids (Majbour et al. 2021; Wang et al. 2015; Graham et al. 2019). Again, since α-syn accumulation is related to an impairment of its metabolism which mainly takes place via the autophagy machinery (Petroi et al. 2012; Limanaqi et al. 2020; Langston and Cookson 2020), autophagy-related proteins were measured in PD patients (e.g., Wu et al. 2011; Miki et al. 2018). This becomes relevant when considering that genetic Parkinsonism mostly involves genes coding for proteins marking specific steps in the autophagy machinery. In fact, a correlation was reported between specific autophagy proteins and PD (Obergasteiger et al. 2018; Pasquali et al. 2009; Limanaqi et al. 2018; Isidoro et al. 2009). However, measurement of single autophagy proteins or even a cluster of autophagy-related proteins out of the cell context and organelles where they operate does not allow to infer confidently with the autophagy status (Klionsky et al. 2021). Thus, it is not surprising that these assay led to inconclusive results. Therefore, in the present study, we analyzed the autophagy machinery using the gold standard procedure, transmission electron microscopy (TEM), which allows to establish the autophagy flux based on the dissection of the ultrastructure of autophagy organelles and proteins within the cell. This investigation was carried from peripheral blood mononuclear cells (PBMC) collected from the blood of PD patients compared with controls. This allows to count a potential ultrastructural pathology of the autophagy machinery in PD compared with healthy volunteers.

The present study was carried out with the aim to improve the power for a potential peripheral marker of PD. In fact, despite assay of specific α-syn isoform in the CSF provides significant differences between PD and controls which may be correlated with some items of the disease course (Majbour et al. 2021), a clear-cut difference between values measured in single PD patients compared with single controls is not present; the same applies for specific autophagy proteins. This means that some overlapping in data distribution exists. Thus, although a significant difference is key in increasing our understanding of the neurobiology of disease, the outcome remains limited in the context of a predictive marker, which should provide information as adherent as possible to the actual damage which produces the disease. In fact, even considering PD as a complex disorder, the loss of integrity in the nigrostriatal innervation should be always be present to validate the diagnosis, and it is ascertained that the loss of nigrostriatal DA innervation below a critical threshold invariably leads to the presence of a movement disorder featuring Parkinsonian symptoms. Likewise, a gold standard marker is expected to possess a similar predictive power. This implies that, in control patients, such a marker is never altered as in PD patients; in turn, it is expected that such a marker in PD patients is always altered to an extent which never occurs in healthy patients. This phenomenon can be defined as a non-overlapping alteration, which clearly discerns PD patients from controls. In this way, the marker does recapitulate the causative pathology/pathobiochemistry of the disease. Such a concept was coined by Hornykiewicz when defining overlapping vs. non-ovelapping pathobiochemical alteration in the brain of PD patients (Hornykiewicz and Pifl 1994). The need for non-overlapping measurement rises up the threshold for accuracy of a reliable PD marker and goes well beyond the occurrence of morphological and biochemical statistical differences.

Thus, this study was focused on the analysis of autophagy status within PBMC cells by an ultrastructural morphometry transmission microscopy approach.

We show a striking difference between PD patients and controls in terms of number of autophagy vacuoles per cell, which, being non-overlapping in the two groups, may be a promising peripheral non-invasive marker.

## Materials and methods

### Subjects

Patients with PD were enrolled among unrelated outpatients referring to the Unit of Neurology of the IRCCS Neuromed form January 2013 to December 2016. The diagnosis of PD was performed according to current diagnostic criteria (Postuma et al. 2015). All of them had been submitted to TC/MRI during the diagnostic workup. Most of them had undergone brain imaging with SPECT with DATscan or ^18^F-DOPA PET during the diagnostic protocol, which was compatible with the clinical diagnosis of PD (Table 1). Neurologically intact subjects were recruited among unrelated caregivers of the patients. Exclusion criteria for being included in the study as unrelated controls were being relatives of patients with neurodegenerative disorders, bearing an altered neurological exam, suffering from any psychiatric disorder. For all subjects included, demographic and clinical data recorded were gender, age, age at disease onset, disease phenotype (tremor-dominant or rigid-akinetic), l-DOPA treatment (yes/not, and dosage treatment at time of PBMC collection). Motor status and motor complications of patients were assessed at the time of PBMC collection by the Unified Parkinson’s Disease Rating Scale (UPDRS) part III during “on” state and “off” state, and by Hoehn and Yahr scale.

**Table 1:** Subjects’ features

	PD subjects	Controls
*N* (males/females)	34 (24/10)	20 (10/10)
Age	61.63 ± 1.62	42.25 ± 2.66
Age at onset	52.53 ± 1.69	–
Disease duration	10.1 ± 7.26	–
MDS-UPDRS part III (on)	18.66 ± 1.03	–
MDS-UPDRS part III (off)	37.07 ± 2.24	–
H&Y	2.7 ± 0.14	–
SPECT/PET availability/total (compatible with PD)	27/30 (27)	–
Average l-DOPA dose (mg/day) (*N* under l-DOPA/total)	517.1 ± 39.45 (28/34)	–

*l-DOPA* l-dihydroxyphenilalanine, *PD* Parkinson’s disease, *PET* positron emission tomography, *SPECT* single-photon emission tomography, *UPDRS part III* Unified Parkinson’s Disease Rating Scale part III (during “on” state and “off” state), *H&Y* Hoehn and Yahr scale

The study protocol was approved by the IRCCS Neuromed, INM Ethics Committee (Protocol ID:CGM-01 Clinical Trials ID:NCT03084224); it was conducted in accordance with the tenets of the Declaration of Helsinki of 1975 and participants or their representatives had given written informed consent for use of their clinical data for research purposes.

### PBMC isolation

Peripheral blood samples (10 mL each) were collected in EDTA vacutainer tubes and PBMCs were isolated from whole blood by Ficoll-Paque PLUS (Sigma-Aldrich, St. Louis, MO, USA). Briefly, blood samples were diluted with the same amount of Phosphate Buffer Saline (PBS), layered on Ficoll-Paque PLUS and centrifuged (2000*g*, 25 min, 15 °C). PBMCs were collected from the interface between plasma and Ficoll-Paque PLUS, washed twice with PBS (2000*g*, 10 min, 15 °C). For each patient, the PBMC fraction was collected and processed for transmission electron microscopy.

### Genetic analysis

Genomic DNA was isolated from peripheral blood leukocytes according to standard procedures (QIAamp DNA Blood Mini Kit—QIAGEN).

#### Multiple ligation-dependent probe amplification (MLPA)

The commercially available kit P051-P052 (MRC-Holland, Amsterdam, Netherlands) was used for the multiplex dosage of exons for the following genes: *TNFRSF9* (1 probe in P051), *DJ1* (4 probes in P051), *ATP13A2* (2 probes in P051, 2 probes in P052), *SNCA* (5 probes in P051, 1 probe in P052), *LPA* (1 probe in P051), *PARKIN* (12 probes in P051, 12 in P052), *LRRK2* (8 probes in P052), *PINK1* (8 probes in P051), *GCH1* (5 probes in P052), *PACRG* (1 probe in P052), *CAV1/2* (2 probes in P052), and *UCHIL1* (4 probes in P052). The MLPA was performed on DNA from patients and four normal subjects were used as internal controls.

#### Genotyping

Genotyping of *PARK1* p.Ala30Pro (c.88G > C, rs104893878), p.Glu46Lys (c.136G > A, rs104893875), p.His50Thr (c.148_149delCAinsAC), p.Ala53Thr (c.157G > A, rs104893877); *PARK8* p.Gly2019Ser (c.6055G > A, rs34637584), p.Arg1441His (c.4322G > A, rs34995376); *GBA* p.Leu444Pro (p.Leu483Pro) (c.1448T > C, rs421016), p.Asn409Ser (p.Asn370Ser) (c. 1226A > G, rs76763715) and *VPS35* p.Asp620Asn (c.1858G > A, rs188286943) SNPs was performed using the MGB-TaqMan Allelic Discrimination method (Applied Biosystems, USA).

The total PCR reaction volume contained 40 ng/μL of genomic DNA, 10 μL of TaqMan master mix II (cat no. 4440043), 0.5 μL 20 × SNP assay mix and was adjusted to a final volume of 20 μL using nuclease free water. The PCR was performed by CFX ConnectTM Real-Time System (Bio-Rad, Hercules, CA, USA), under the following conditions: initial enzyme activation at 95 °C for 10 min, followed by 40 cycles of amplification; denaturation at 95 °C for 15 s, annealing/extension for 1 min at 60 °C. Fluorescence data collection was performed at annealing/extension step for FAM and VIC dye.

#### Clinical exome

Clinical exome sequencing considering roughly 5000 human genes (this analysis including 17 genes related to Parkinson disease: *PARK1:SNCA; PARK2:PRKN; PARK3:SPR; PARK5:UCHL1; PARK6:PINK1; PARK7:DJ1; PARK8:LRRK2; PARK9:ATP13A2; PARK10:ELAVL4; PARK11:GIGYF2; PARK12:TAF1; PARK13:HTRA2; PARK14:PLA2G6; PARK15:FBXO7; PARK16:ADORA1; PARK17:VPS35; PARK18:EIF4GI*) was performed using the Clinical Exome Solution kit (Sophia Genetics, SA, Boston, MA, USA), following the manufacturer’s instructions. The resulting libraries were processed for paired-end sequencing on the MiSeq platform Illumina (San Diego, CA, USA). Sophia DDM^®^ platform (Sophia Genetics, SA) was used for automated annotation, characterization, and selection of potentially pathogenic variants. Direct evaluation of the data sequence was performed by the Integrative Genomics Viewer v.2.3.

A second analysis using GenomeUp platform was performed (https://platform.genomeup.com/) using the Best Practices workflows of GATK v4.1 for germline variant calling.

Potentially pathogenic variants were interpreted according to ACMG criteria (Richards et al. 2015). ACMG classification was compared with automatic classification performed by Varsome genome interpreter (https://varsome.com/).

### Transmission electron microscopy

For TEM analysis, PBMC were fixed by adding a fixing solution (2.0% paraformaldehyde/0.1% glutaraldehyde, both dissolved in 0.1 M PBS pH 7.4) for 90 min at 4 °C.

After washing, fixed PBMC specimens were post-fixed in 1% OsO_4_ for 1 h at 4 °C and then dehydrated in ethanol to be finally embedded in epoxy resin. For ultrastructural analysis, grids containing non-serial ultrathin sections (40–50 nm thick) were examined at TEM, at a magnification of 8,000x; for each subject, several grids were analyzed to count a total number of 100 cells for subject.

Ultrathin sections were stained with uranyl acetate and lead citrate, and they were finally examined using a JEOL JEM-100SX transmission electron microscope (JEOL, Tokyo, Japan).

#### Post-embedding immunocytochemistry

Plain TEM was implemented by a post-embedding immunocytochemistry with primary antibodies against Microtubule-associated proteins 1A/1B light chain 3B (LC3), to explore autophagy according to the manuscript “Guidelines for the Use and Interpretation of Assays for Monitoring Autophagy (4th Edition)” (Klionsky et al. 2021), or with primary antibodies anti-α-syn.

Fixing and post-fixing solutions as well as epoxy resin were validated in previous studies for immuno-gold-based ultrastructural morphometry (Bendayan and Zollinger 1983; Lenzi et al. 2012; Lazzeri et al. 2018).

Post-embedding procedure was carried out on ultrathin sections collected on nickel grids, which were incubated on droplets of aqueous sodium metaperiodate (NaIO_4_), for 30 min, at room temperature to remove OsO_4_. NaIO_4_ is an oxidizing agent allowing a closer contact between antibodies and antigens by removing OsO_4_ (Bendayan and Zollinger 1983).

Grids were washed in PBS and incubated in a blocking solution containing 10% goat serum and 0.2% saponin for 20 min, at room temperature. For immune-cytochemistry, for LC3, they were incubated with a primary antibody solution containing rabbit anti-LC3 (Abcam, Cambridge, UK, diluted 1:50) with 0.2% saponin and 1% goat serum in a humidified chamber over-night, at 4 °C. After washing in PBS, grids were incubated with secondary anti-rabbit antibodies conjugated with gold particles (10 nm mean diameter, BB International, Crumlin, UK), which were diluted 1:30 in PBS containing 0.2% saponin and 1% goat serum for 1 h, at room temperature.

The same protocol was used for α-syn immuno-cytochemistry. Mouse anti-α-syn (Abcam, diluted 1:100) and secondary anti-mouse antibody conjugated with gold particles (20 nm mean diameter, BB International, diluted 1:80) were used. Sections working as methodological control were incubated with secondary antibody only. After incubation with secondary antibody and PBS washing, grids were incubated with droplets of 1% glutaraldehyde for 3 min; then grids were washed with droplets of distilled water to prevent salt traces and precipitation of uranyl acetate.

#### Ultrastructural morphometry

Transmission electron microscopy was carried out at 8000 × magnification to analyze cell compartments (Lucocq et al. 2004; Lazzeri et al. 2018), concomitantly with immuno-gold particles. To scan the whole cell pellet within each grid square, counts were started from a corner of a randomly identified grid square. Autophagy vacuoles were identified by the gold standard technique, TEM, as vacuoles surrounded by a single, double, or multiple membrane, owing an electron density, which is comparable to surrounding cytosol staining for LC3 according to Klionsky et al. (2021). For each cell, the number of autophagosomes, LC3 and a-syn particles stoichiometrically stained by immune-gold were counted.

### Statistical analysis

Statistical analysis was carried out by StatView software. Data are reported as the mean ± SEM per cell.

After verifying a normal distribution of the three parameters assessed (autophagy vacuoles, LC3 and α-syn particles per cell) comparisons among different groups were carried out by student’s *t* student for unpaired data. Correlation analysis between subject’s data was performed by calculating Pearson’s coefficient. Bonferroni correction for multiple comparisons was applied to the statistical analysis. The null hypothesis (*H*_0_) was rejected for *P* ≤ 0.05.

## Results

### Subjects’ features

The clinical features of the subjects enrolled in the study are reported in Table 1. In particular, PD was tremor-dominant in one third of subjects and rigid-akinetic the remaining ones. All PD patients had been submitted to genetic testing; in six of them, it was shown a genetic alteration in loci associated with genetic PD. In detail, two patients, affected by familial PD, own a triplication of the SNCA gene, while one patient own a familial mutation of PARK13 locus. Mutations were found also in three sporadic PD patients carrying a mutation of Grb10-Interacting GYF Protein 2 (GIGYF2) gene, the LRKK2 gene, and a duplication of exons 2–3 of PARK2 gene. At the time of enrollment, the mean age of patients was 61.6 ± 1.62, and while in controls, it was 42.25 ± 2.66 (*P* < 0.001). Despite such an age difference, this was not significantly affecting data within each group as analyzed later in the manuscript.

Twenty-eight subjects were under treatment with L-DOPA at the time of PBMC collection; mean L-DOPA daily dosage was 517.1 ± 39.45 mg. Six subjects had not received yet any PD medication at time of PBMC collection and were analyzed as de novo sub-population.

### Autophagy vacuoles content in PBMC

**Fig. 1:**
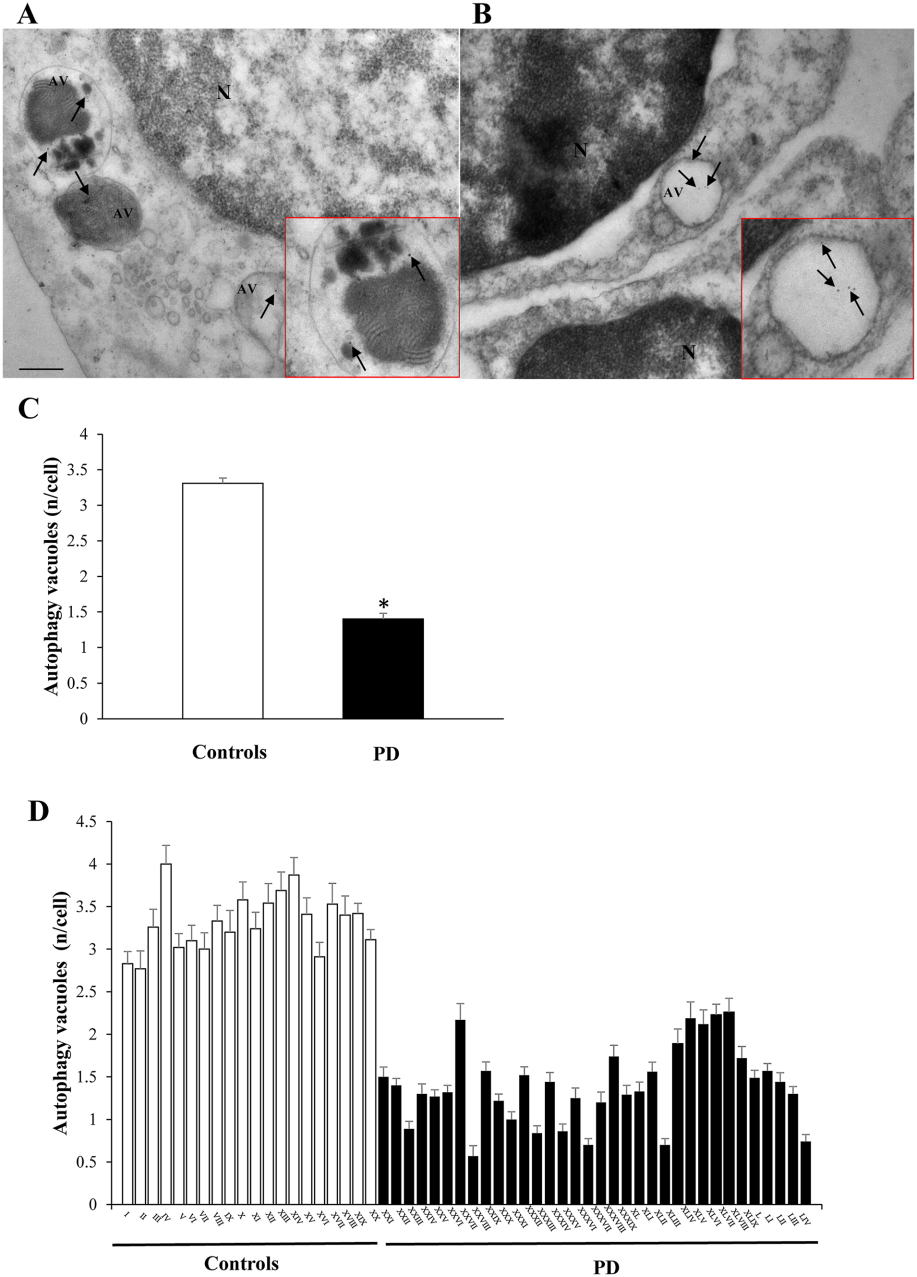
Autophagy vacuoles in PBMC of PD patients and controls. Representative transmission electron microscopy picture of PBMC from a control (**A**) and a patient affected by idiopathic PD (**B**). Arrows point to LC-3-immuno-gold particles (10 nm mean diameter) within autophagy vacuoles (AV), which are represented by single/multiple membrane vacuoles possessing the same electron density of the surrounding cytoplasm. Insert within each picture shows a higher magnification of LC3-positive immune-gold particles within vacuoles. Graph **C** reports the mean values for controls and PD patients: in PD subjects there is a significantly lower amount of autophagy vacuoles/cell compared with controls. In graph **D,** the values are reported for each single subject of the two groups; in none of PD subjects, there are a number of autophagy vacuoles comparable with the lowest number observed among controls. Counts represent the mean ± S.E.M from *N* = 100 cells per group. *P < 0.05 compared with controls. Lower magnification scale bar 200 nm. Higher magnification (insert) scale bar 100 nm. *N* nucleus

In the PBMC of the whole PD population, there was a significant decrease in the mean number of autophagy vacuoles per cell compared with controls (1.40 ± 0.08 and 3.31 ± 0.07, respectively; *P* = 0.0006) (Fig. 1). Most importantly, in any PD patient, the mean number of PBMC autophagy vacuoles was ≥ than in any control subject (highest mean value among patients: 2.26 (# XLVIII, Fig. 1), lowest value among controls: 2.77 (# II, Fig. 1). The difference between PD and controls in the mean number of autophagy vacuoles per cell was statistically significant also when considering only PD patients without genetic alterations (1.42 ± 0.08; *P* < 0.0001 vs controls). The mean PBMC content of autophagy vacuoles did not correlate with disease duration, which was measured in the whole group of subjects under treatment (*r* = − 0.50; *P* = 0.018), and in the group of PD patients under treatment without including those affected by gene alterations (*r* = − 0.52; *P* = 0.038). Conversely, this was not the case when considering the whole group of patients, including newly diagnosed de novo patients (*r* = − 0.37; *P* = 0.1). No correlation was measured between number of vacuoles and age, neither within PD group, nor in control group. Similarly, within PD patients, the mean number of vacuoles did not correlate with disease severity, neither using UPDRs-on, nor with UPDRs-off, or with H&Y rating scales.

#### *Autophagy vacuoles in *de novo* PD subjects*

Despite the small sample, it is remarkable that even in those patients, who were just diagnosed PD, and who never received any DA substitution therapy, so-called de novo, the number of autophagy vacuoles never overlaps with values counted in controls. The mean was even below that measured within the whole PD population (1.26 + 0.18 and 1.42 ± 0.08, respectively). The number of LC3 (77.42 ± 13.11) and α-syn particles (5.99 ± 1.39) did not differ from in-treatment PD patients.

### α-Syn ultrastructural stoichiometry

**Fig. 2:**
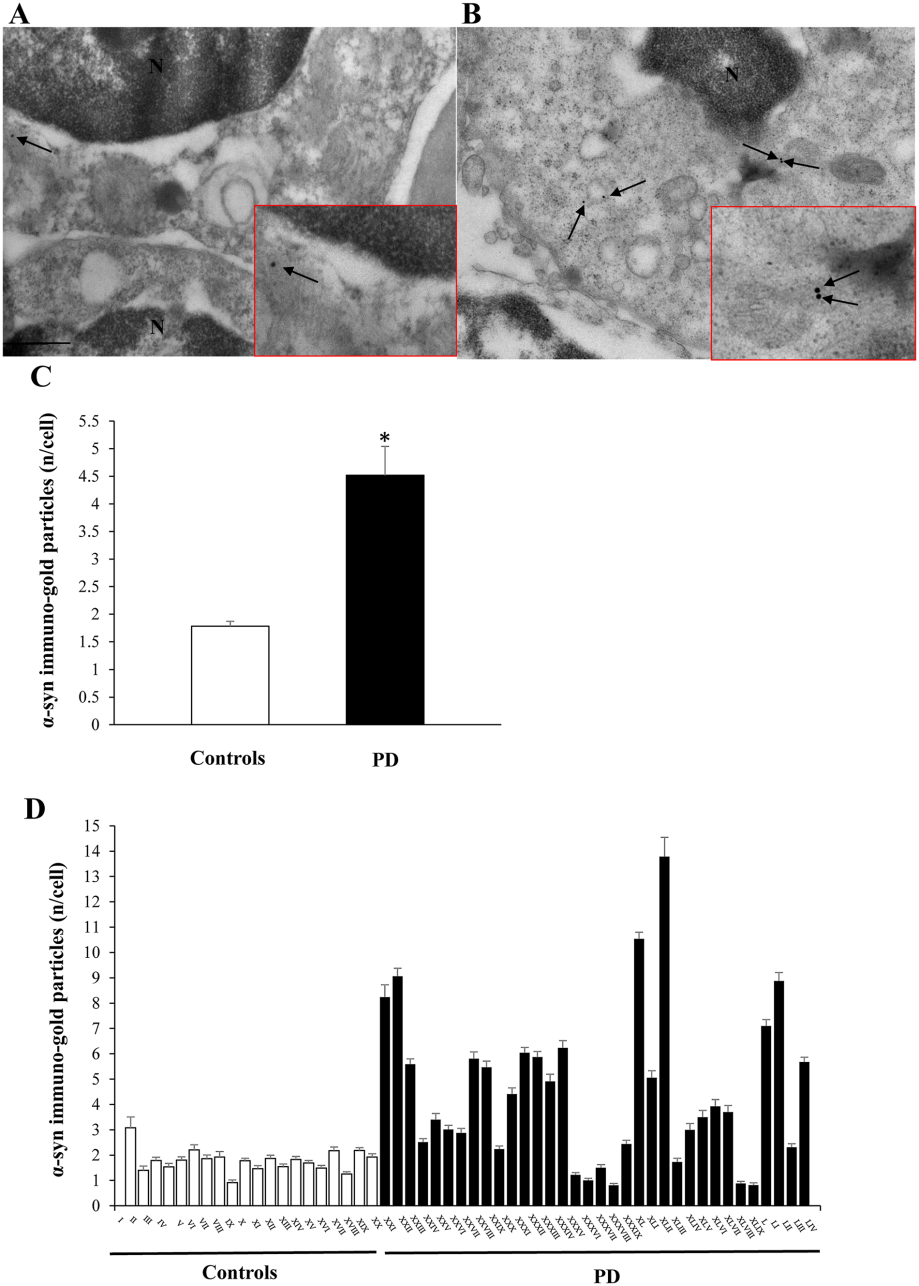
α-Syn ultrastructural stoichiometry in PBMC of PD patients and controls. Representative transmission electron microscopy pictures of PBMC from a control (**A**) and a patient affected by idiopathic PD (**B**). Arrows point to α-syn immuno-gold particles (20 nm mean diameter) dispersed within the cytosol. Insert within each plate, shows a higher magnification of α-syn immuno-gold particles. Graph **C** reports the mean values of PBMC α-synuclein immuno-gold particles/cell for controls and PD patients: in PD subjects there is a significantly higher amount of particles/cell compared with controls. In graph **D,** the mean values are reported for each single subject of the two groups; many of PD subjects show a higher number of α-synuclein immune-gold particles than controls, but some controls show a number of immune-gold particles higher than that found in selected PD subjects. Counts represent the mean ± S.E.M from *N* = 100 cells per group. *P < 0.05 compared with controls. Lower magnification scale bar 200 nm. Higher magnification (insert) scale bar 100 nm. *N* nucleus

The number of α-syn immuno-gold particles within PBMC of PD patients (4.52 ± 0.05) was significantly higher (*P* < 0.0001) compared with controls (1.78 ± 0.44) (Fig. 2). Such a difference was confirmed even when solely considering PD patients without genetic alterations (4.07 ± 0.49 α-syn particles/cell, *P* = 0.0034 compared with controls). In PD patients, α-syn content within PBMC did not correlate neither with disease severity at time of blood collection (when assessed by UPDRs-on, or with UPDRs-off, or with H&Y score), nor with disease duration. This occurs when considering the whole group of PD patients, non-genetic PD patients or PD patients under treatment. Furthermore, there was no correlation of PBMC α-syn content with age, neither in patients nor in control subjects. Differing from what observed for autophagy vacuoles, the number of α-syn particles/cell was overlapping between controls and patients (Fig. 2). As expected, in patients carrying a duplication/triplication of *SNCA* gene, *PARK4*, (# XX1 and # XXII) a high content of α-syn particles per cell (8.24 ± 0.48, and 9.06 ± 0.32, respectively) was measured. Nonetheless, the patient carrying a mutation of *PARK13* (*LRKK2*) owns higher levels of α-syn (10.54 ± 0.25).

### LC3 ultrastructural stoichiometry

**Fig. 3:**
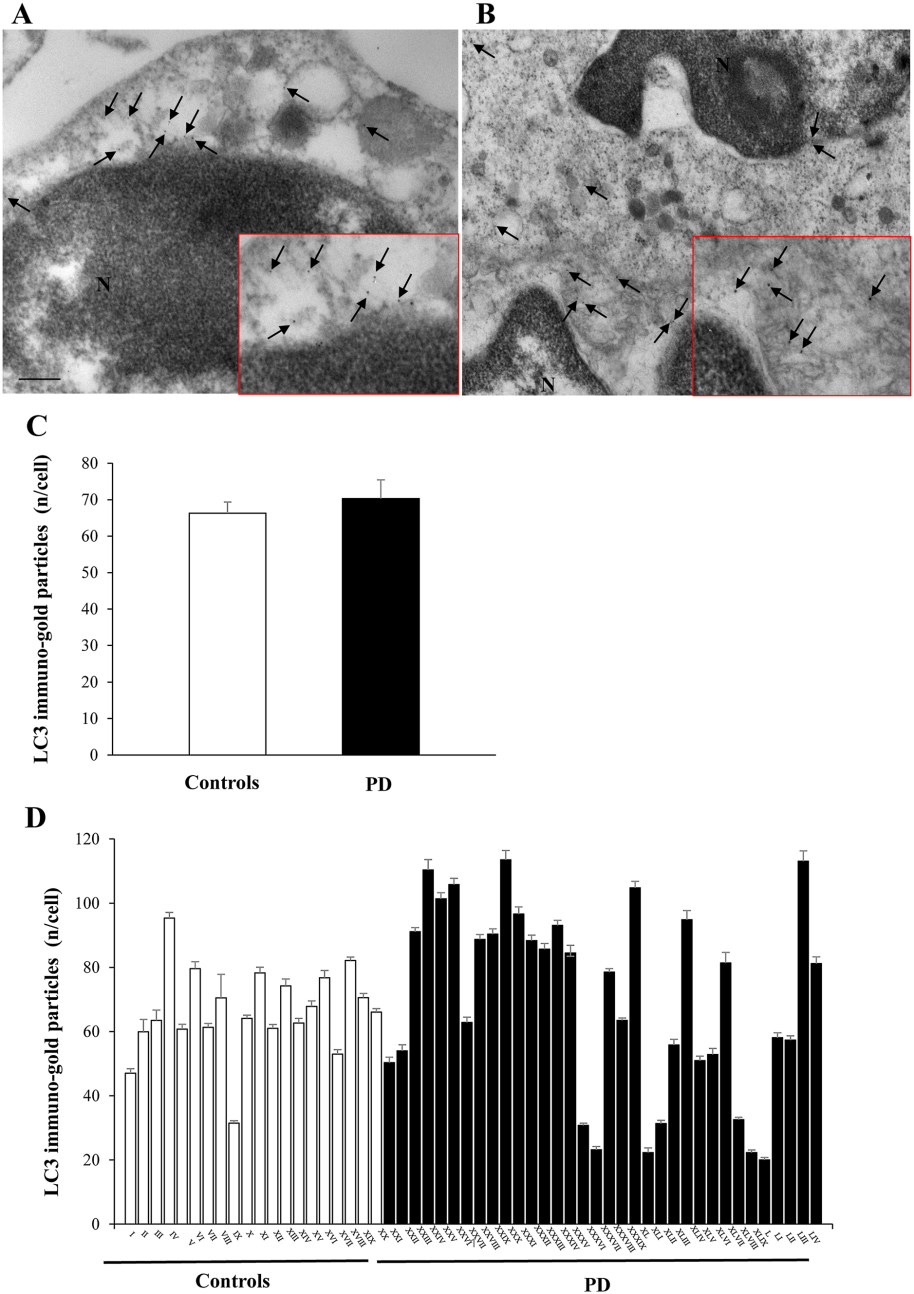
LC3 ultrastructural stoichiometry in PBMC of PD patients and controls. Representative transmission electron microscopy picture of PBMC from a control (**A**) and a patient affected by idiopathic PD (**B**). Arrows point to LC3 immuno-gold particles (10 nm mean diameter) dispersed within the cytosol. Insert within each plate, shows a higher magnification of LC3 immuno-gold particles. Graph **C** reports the mean values of PBMC LC3 immuno-gold particles/cell for controls and PD patients: the two groups do not show statistically significant differences. In graph **D**, the mean values are reported for each single subject of the two groups. Counts represent the mean ± S.E.M from *N* = 100 cells per group. *P < 0.05 compared with controls. Lower magnification scale bar 200 nm. Higher magnification (insert) scale bar 100 nm. *N *nucleus

LC3 was not significantly different between PD patients and controls (mean 70.32 ± 5.12, and 66.30 ± 3.05, respectively) (*P* = 0.47) (Fig. 3); this was confirmed in the group of PD patients without genetic alterations (mean LC3 particles/cell, 70.06 ± 4.99). Moreover, neither in PD patients nor in controls, the number of LC3 particles correlates with age.

## Discussion

In this study, TEM was used as a gold standard to assess and count specific ultrastructural morphometry to measure the organelle autophagosome within PBMC of PD patients and controls. The study was carried out based on several data showing the involvement of the autophagy pathway in the pathophysiology of PD. The investigation was carried out in the hope to refine the measurement of autophagy-related subcellular structures in PD patients to disclose some disease-specific alterations. The present study was moved by the need to dissect a potential marker, which clearly distinguishes PD patients from controls through a deeper investigation of autophagy-related structures. The occurrence of autophagy vacuoles in PBMC from PD patients is significantly lower than controls; most remarkably, such a difference so far is non-overlapping, which matches the major need for a disease marker, being the number of autophagy counted in each PD patient always much lower than those counted in the control subject owing the lowest amount of autophagy vacuoles. Such a non-overlapping clear-cut distinction is promising and it needs to be validated in large populations on a wide range of patients. A number of bias need to be considered. In fact, DA substitution therapy is expected to alter peripheral markers of autophagy; however, when counted in de novo patients, the mean number of autophagy vacuoles was even lower compared with the whole PD population. This suggests that therapy may not play a role in the outcome of this study. Since age might play a role in autophagy, and age is different between the two groups, we measured whether age differences were responsible for a change in the number of autophagosomes. No age correlation was statistically detected in the group of controls between age and the number of autophagosomes. Even considering the PD group, which features an older age, compatible with an autophagy impairment, the marked decrease in the number of autophagosomes is not related with the age of PD patients. By incidence, the number of autophagy vacuoles in the youngest PD subjects was lower and it was never overlapping with the oldest subjects of the controls’ group.

The present research study indicates that suppressed number of autophagy vacuoles may be validated as a pathological peripheral marker in PD. In detail, autophagy vacuoles were positively assessed within PBMC and their number was counted following a stoichiometric analysis of LC3-positive vacuoles according to the Guidelines to monitor autophagy (Klionsky et al. 2021). In PD patients, autophagy vacuoles are significantly reduced compared with those measured in neurologically intact controls. In parallel analysis, stoichiometric counts of α-syn within these cells were increased significantly in PD. However, the increase in α-syn, despite its significance, provided an overlapping distribution, where some PD patients possessed lower α-syn compared with some controls. A number of studies measured α-syn levels from the blood and CSF. This was carried out also aiming at specific isoforms of α-syn (monomer vs. oligomers Miki et al. 2018; Majbour et al. 2021; soluble vs. insoluble Prigione et al. 2010), which best characterize PD. The present study confirms a significant increase of α-syn levels, here measured using ultrastructural stoichiometry evidence. These findings contribute to improve our understanding about the neurobiology of PD, although this remains an overlapping difference, which does not seem to work as a clear-cut marker for a reliable diagnosis of PD.

While getting these data, one major issue we thought as a confounding bias was the role produce by DA substitution therapy. Despite the analysis carried out in de novo patients makes this hypothesis unlikely, such a topic deserves further considerations. In fact, it is known that, within the CNS, DA may increase the number of autophagy vacuoles in target cells (Lazzeri et al. 2018). Therefore, to explore the potential effect of PD treatment on the autophagy status assessed here, the study was extended to some de novo PD patients. Promisingly, all de novo patients possess a number of autophagy vacuoles in the same range of the whole PD group, which is below each control. Even in this case, we expect to validate these findings through the study of a high number of de novo PD patients. To our knowledge, the issue of therapy in conditioning the expression of autophagy-related structures within PBMC from PD was never considered so far. Previous studies measuring autophagy markers in PBMC from PD patients, were carried out in PD subjects under treatment, which in most cases is L-DOPA, as it is in the present group of patients.

The issue of genetic Parkinsonism is confined only to 6 patients, one *PARK2*, two *PARK4*, one *PARK8,* one *PARK 13* and an additional variant of GRB10 interacting GYF protein 2, as reported in the Results section. It is remarkable that even in this small group, the number of autophagosomes remains in the same range of the whole group of PD patients, despite a significant difference concerning disease severity, and the high amount of α-syn detected in two patients affected by α-syn multiplication (*PARK4*). The measurement of an excess of α-syn way exceeding other patients in siblings carrying *α-SYN* gene multiplication represents an inner validation for the reliability of the ultrastructural stoichiometric measurement carried out in the present work. This quantitative value incidentally provides an accurate measurement of how much more protein is produced by such a gene multiplication.

The method used here, despite being sophisticated concerning the ultrastructural approach and morphometric stoichiometry (ultrastructural morphometry), provides a feasible non-invasive test when applied routinely. In detail, we analyzed subcellular autophagy compartment from PBMC of PD and non-PD patients. Blood cells ultrastructure was already reported as a significant marker in Huntington’ disease (Squitieri et al. 2010), while other studies assayed autophagy protein from these cells without ultrastructural analysis (Wu et al. 2011; Miki et al. 2018; Papagiannakis et al. 2019). The present study is the first to document blood cell ultrastructure of autophagy-related organelles and proteins. The main finding of the present work consists in counting a number of autophagy vacuoles in PD patients, which is always lower than the lowest number measured in controls. Such a non-overlapping count is promising as a potential marker and it deserves to be validated in a large number of patients and controls, where stratified measurements (symptoms, progression, severity, disease duration, genetics), may disclose additional correlations we do not report here in order not to over-interpreting beyond the main finding. Moreover, it will be fascinating to assess whether these findings apply to other neurodegenerative disorders clustering a specific disease group (such as synucleinopathies) or more widespread, or instead being specific for Parkinsonian syndromes.

Autophagy is a ubiquitous cell mechanism, which is key for the degradation of pathological proteins, as well as for the proper cell homeostasis and organelles recycling (Dikic and Elazar 2018). Idiopathic PD has been repeatedly shown to be associated with an altered autophagy, dating back to the early pathological study by Anglade et al. (1997) in the substantia nigra of PD patients. Several studies have confirmed an alteration of autophagy in PD also in parallel with increased α-syn, which is likely to depend on the autophagy alteration itself (Chu et al. 2009; Dehay et al. 2010; Klucken et al. 2012). Peripheral blood mononuclear cells have been already used to measure autophagy-related RNA and/or proteins as potential biomarkers in patients affected by PD, although conflicting results exist (Miki et al. 2018; Papagiannakis et al. 2019; Prigione et al. 2010). This is confirmed by the inconclusive findings obtained here when measuring the autophagy-related protein LC3.

The reduction of autophagy vacuoles in the PBMC of PD patients measured in the present study is in line with the findings of some of those studies in PBMC in PD. In particular, Miki et al., in 2018 assessed the expression of mRNA for different genes involved in autophagy in PD patients, and control subjects and showed a decrease of these in PD. Similarly, Papagiannakis et al. (2019) showed that in PD patients, there is a significant reduction of autophagy proteins, in parallel with a decrease of lysosomal degradation in cultured PBMC cells.

Several proteins co-operate to the proper functioning of autophagy. LC3 is considered as a marker of autophagy; however, its increase can be related also to a reduced progression of autophagy from autophagosome to autophagolysosome (Klionsky et al. 2021). Thus, a change in the amount of LC3 is not predicting the autophagy status, and it is not relevant for measuring autophagy being potentially witnessing a decrease, an increase or no change in the efficacy of autophagy. In fact, some works measured an increase (Wu et al. 2011; Prigione et al. 2010), while others, a lack of significant modification (Miki et al. 2018) of LC3 levels in PBMC from PD patients. It is likely that reduced LAMP-2 gene and protein expression (Wu et al. 2017) owns further significance in witnessing reduced autophagy status in PD. Thus, further studies are in progress with the aim at first to validate the autophagosome suppression as a predictable disease marker, as well as to ascertain its role in the biology of PD. This latter point could include the measurement of LAMP-2.

The reasons explaining an autophagy alteration in peripheral blood cells in a CNS disorder are currently unknown. A fascinating explanation might involve exosomes. The latter are extracellular vesicles within the nanometer diameter range, which are released in the extracellular matrix by different cell types, including neurons, and can concur to cell-to-cell spreading altered proteins (Colombo et al. 2014). Exosomes produced within the CNS can cross the blood–brain barrier and reach out the blood (Maurella et al. 2015). This is documented in PD patients (Jiang et al. 2020; Leng et al. 2020), in which a variety of exosomes have been demonstrated (Wu et al. 2017). Thus, an intriguing hypothesis to explain the occurrence of autophagy impairment in PBMC in PD is the chance that exosomes may spread suppression of autophagy machinery. This is an interesting matter for future investigations.

In conclusions, the present study indicates that, in a small population, the number of autophagyvacuoles, measured within PBMC, highly discriminates healthy controls from patients affected by PD (including genetic Parkinsonism). Apart from being significantly reduced in PD patients compared with controls, the number of autophagosomes in each PD patient is always lower than each control patient (non-overlapping alteration). When confirmed this may provide a reliable, non-invasive marker in PD to be extended to other degenerative disorders.

## Data Availability

A repository with all rough data is available upon request to the authors.
